# Supplementary material for: Baseline IgM Amounts Can Identify Patients with Poor Outcomes: Results from a Real-Life Single-Center Study on Classical Hodgkin Lymphoma
Source: Cancers (Basel). 2024 Feb 18;16(4):826. doi: 10.3390/cancers16040826 (PMC10886525; doi:10.3390/cancers16040826)

Supplementary Table 1: clinical characteristics of patients included in the training and validation sets

	**All patients (n=212)**	**Training set (n=106)**	**Validation set (n=106)**	**p**
**Age <45 years, n (%)**	148 (69.8%)	75 (63.3)	73 (68.9)	0.99
**Gender male, n (%)**	109 (51.4%)	56 (52.8)	53 (50%)	0.99
**Median WBC, cells/mmc (range)**	9.86 (1.12-26.00)	9.75 (1.12-25.80)	10.05 (2.20-26.00)	0.99
**Median ANC, cells/mmc (range)**	7.24 (0.79-22.20)	7.07 (0.79-20.60)	7.29 (0.80-22.20)	0.99
**Median ALC, cells/mmc (range)**	1.46 (0.19-3.95)	1.45 (0.19-3.95)	1.50 (0.19-3.56)	0.99
**Median NLR, cells/mmc (range)**	4.79 (0.60-64.7)	4.81 (1.18-53.40)	4.73 (0.60-64.7)	0.99
**Presence of large nodal mass, n (%)**	64 (30.2%)	37 (34.9)	27 (25.5)	0.74
**Presence of extranodal sites, n (%)**	53 (25.0%)	26 (24.5)	27 (25.5)	0.96
**IPS score ≥3, n (%)**	54 (25.5%)	26 (24.5)	28 (26.4)	0.92
**IgM <=50mg/dL, n (%)**	49 (23.1%)	23 (21.7)	26 (24.5)	0.86
**Positive PET-2, n (%)**	33 (15.6%)	19 (17.9)	14 (13.2)	0.85

Supplementary Table 2: univariate analysis of PFS in the training set

Clinical variable	N	5-years PFS	p-value	HR	95% CI
**Age**					
≤ 45 years	75	66.2%	0.65	0.8	0.4-1.8
> 45 years	31	74.9%			
**Gender**					
Male	56	66.3%	0.9	0.99	0.5-1.9
Female	50	65.1%			
**WBC**					
<15.000 cells u/L	88	71.6%	0.13	2.1	0.8-5.4
≥15.00 cells u/L	18	55.1%			
**ALC**					
<600 cells u/L	10	57.1%	0.54	1.4	0.5-4.8
≥600 cells u/L	96	69.7%			
**Bulky disease**					
Absent	69	71.6%	0.25	1.3	0.7-3.1
Present	37	63.0%			
**Extranodal disease**					
Absent	80	74.5%	***0.03***	2.7	1.2-6.4
Present	26	50.2%			
**IPS**					
<3	80	73.4%	***0.01***	2.9	1.3-7.1
≥3	26	52.7%			
**NLR**					
<6	63	81.3%	***0.0001***	4.4	2.1-9.1
≥6	42	47.8%			
**IgM**					
≤50 mg/dL	23	54.9%	***0.02***	2.8	1.2-6.6
>50 mg/dL	83	72.5%			
**PET2-status**					
Negative	87	77.6%	***<0.0001***	21.6	7.4-63.5
Positive	19	26.2%			

Supplementary Table 3: univariate analysis of PFS in the validation set

Clinical variable	N	5-years PFS	p-value	HR	95% CI
**Age**					
≤ 45 years	73	80.6	0.4	1.5	0.6-3.8
> 45 years	33	72.9			
**Gender**					
Male	53	68.0	***0.03***	2.7	1.1-6.5
Female	53	87.9			
**WBC**					
<15.000 cells u/L	90	81.5	***0.01***	5.4	1.4-20.9
≥15.00 cells u/L	16	60.6			
**ALC**					
<600 cells u/L	101	79.7	0.13	5.8	0.6-59.7
≥600 cells u/L	5	53.3			
**Bulky disease**					
Absent	79	83.0	***0.04***	2.8	1.0-7.6
Present	27	65.0			
**Extranodal disease**					
Absent	79	80.8	0.24	1.8	0.6-5.1
Present	27	70.4			
**IPS**					
<3	78	82.0	0.1	2.3	0.8-6.5
≥3	28	67.1			
**NLR**					
<6	64	78.2	0.94	1.0	0.4-2.5
≥6	42	78.7			
**IgM**					
≤50 mg/dL	26	53.8	***0.0001***	11.0	3.7-32.6
>50 mg/dL	80	86.6			
**PET2-status**					
Negative	92	86.3	***0.0001***	193	40-922
Positive	14	28.6			

Supplementary Figure 1

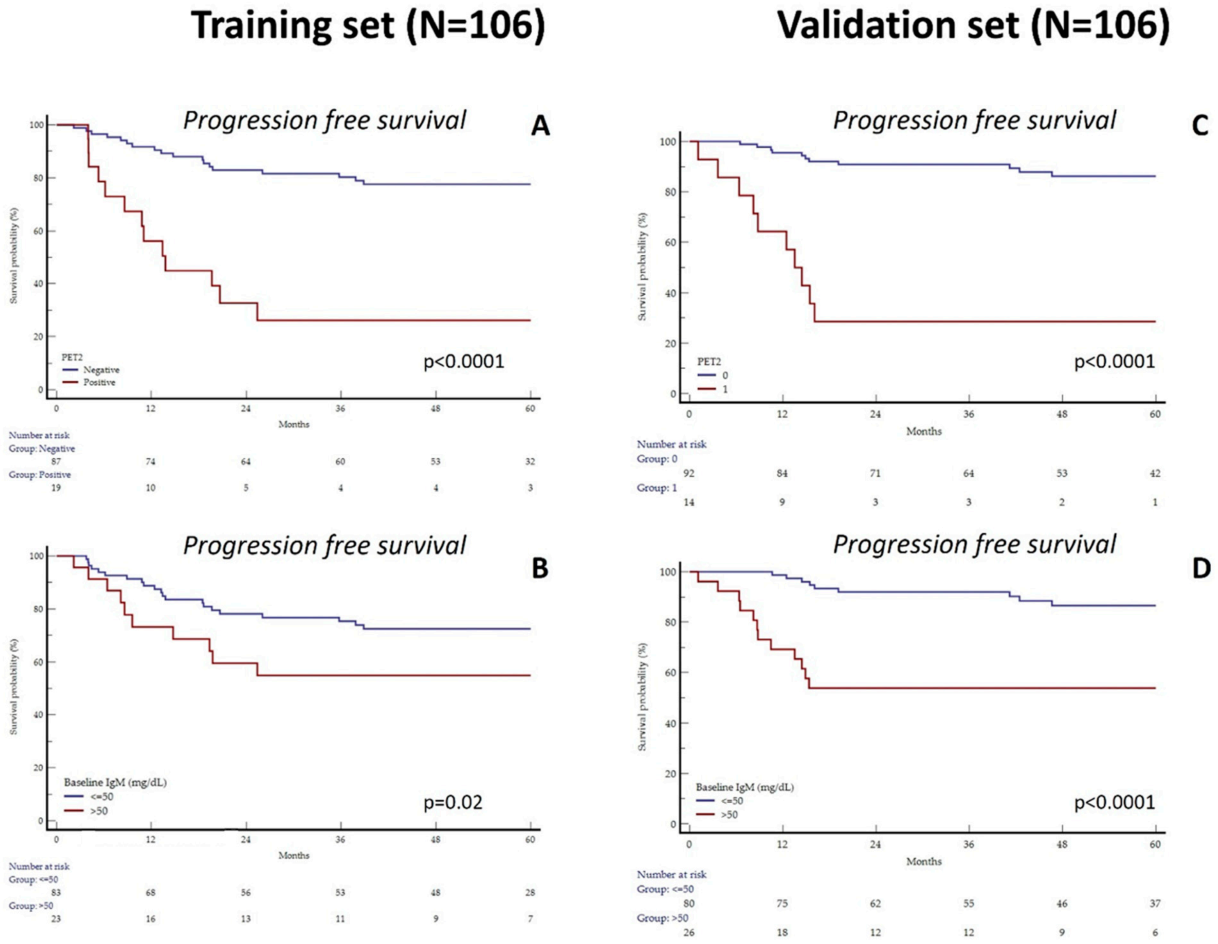

Supplement: Supplementary file 1 [file cancers-16-00826-s001.zip › cancers-2847458-SI.pdf]
